# The neuropeptide calcitonin gene-related peptide links perineural invasion with lymph node metastasis in oral squamous cell carcinoma

**DOI:** 10.1186/s12885-021-08998-9

**Published:** 2021-11-20

**Authors:** Yu Zhang, Mingtao Chen, Zheqi Liu, Xu Wang, Tong Ji

**Affiliations:** 1 Department of Oral Maxillofacial-Head Neck Oncology, Shanghai Ninth People’s Hospital, Shanghai Ninth People’s Hospital, Shanghai Jiao Tong University School of Medicine, Shanghai, 200011 China; 2 College of Stomatology, Shanghai Jiao Tong University, Shanghai, 200011 China; 3 National Center for Stomatology, National Clinical Research Center for Oral Diseases, Shanghai, 200011 China; 4 Shanghai Key Laboratory of Stomatology, Shanghai, 200011 China

**Keywords:** Oral squamous cell carcinoma, Perineural invasion, Lymph node metastasis, Calcitonin gene-related peptide

## Abstract

**Objective:**

Although perineural invasion (PNI) is well-known to be correlated with and able to predict lymph node metastasis (LNM) in oral squamous cell carcinoma (OSCC), the clinical and molecular correlation between PNI and LNM has not been elucidated, and preoperative biomarkers for LNM prediction in OSCC are urgently needed.

**Materials and methods:**

The correlation between PNI and LNM was retrospectively evaluated using a cohort of 218 patients diagnosed with OSCC. Candidate neuropeptides were screened based on TCGA database and verified via immunohistochemistry and Western blot analyses. ELISA was used to detect calcitonin gene-related peptide (CGRP) in patient plasma. In vitro assays were used to explore the effects of CGRP on OSCC cells.

**Results:**

OSCC patients with PNI had a higher incidence of LNM (69.86% vs. 26.2%, *P* < 0.0001, *n* = 218). CGRP expression was upregulated in the PNI niche and in metastatic lymph nodes, and was correlated with poor overall survival of OSCC patients. Preoperative plasma CGRP levels were higher in OSCC patients (*n* = 70) compared to healthy donors (*n* = 60) (48.59 vs. 14.58 pg/ml, *P* < 0.0001), and were correlated with LNM (*P* < 0.0001) and PNI (*P* = 0.0002). Preoperative plasma CGRP levels alone yielded an AUC value of 0.8088 to predict LNM, and CGRP levels combined with preoperative T stage reached an AUC value of 0.8590. CGRP promoted proliferation and migration abilities of OSCC cells, which could be antagonized by either pharmacological or genetic blockade of the CGRP receptor.

**Conclusions:**

The neuropeptide CGRP links PNI and LNM in OSCC, and preoperative plasma CGRP levels can be used to predict LNM in OSCC.

**Supplementary Information:**

The online version contains supplementary material available at 10.1186/s12885-021-08998-9.

## Introduction

Oral cancer is a common malignant tumor that occurs in the oral epithelial tissue, and more than 90% of cases of oral cancer are oral squamous cell carcinoma (OSCC) [1]. One of the most prominent characteristics of OSCC is lymph node metastasis (LNM), even in early-stage disease, which is one of the most significant factors for patient prognosis [2–4]. Many studies suggested that elective neck dissection is recommended for early stage OSCC [2]. However, nearly 70% of early stage OSCC patients undergo unnecessary neck dissection [5]. The sentinel lymph node biopsy has aroused great attention for its considerable sensitivity to detect metastasis in lymph nodes during surgery, thus helping surgeons make personalized operation plan [6, 7]. Many researchers now have used molecular biomarkers for LNM prediction. However, most of these biomarkers are based on mRNA or protein expression in tumor tissues [8–10], so are impractical for preoperative prediction. Therefore, preoperative biomarkers for LNM prediction that can be measured non-invasively are urgently needed.

Another important clinicopathological feature of OSCC is perineural invasion (PNI), which has been considered an adverse feature of head and neck cancers in the National Comprehensive Cancer Network guidelines since 2017 [11, 12]. In addition to the clear negative effects of PNI on outcome in OSCC patients [13–15], PNI status has been reported to be correlated with LNM in many studies [16–18]. A study published recently in *Cell* demonstrated that lymph nodes are innervated by a unique population of sensory nerves [19], indicating a potential anatomical connection between PNI and LNM based on the abundant innervation of the oral cavity. Although some studies found that PNI could be used to predict LNM in OSCC [17], PNI status can only be precisely determined through pathological diagnosis based on tumor tissues, which limits its clinical use. Elucidating the molecular connection between PNI and LNM can thus help to understand their clinical correlation and develop preoperative biomarkers to predict LNM in OSCC patients.

Calcitonin gene-related peptide (CGRP) is often used as a marker of nociceptive nerves and is the most abundant neuropeptide in trigeminal nerves [20, 21]. Several studies have reported that CGRP can directly function on various types of cancer like metastatic breast cancer [22], prostate cancer [23] and osteosarcoma [24]. Moreover, endogenous CGRP can promote cancer progression through facilitating tumor-associated angiogenesis [25]. However, the role of CGRP in OSCC and its correlation with PNI and LNM remain unknown.

In the present study, the correlation between PNI and LNM was retrospectively explored using a cohort of 218 patients diagnosed with OSCC. CGRP was screened and verified as the molecular link between PNI and LNM through TCGA IHC and Western blot analyses. The predictive relevance of preoperative plasma CGRP levels was determined through receiver operating characteristic (ROC) analysis. Finally, in vitro assays were used to explore the effects of CGRP on OSCC cells.

## Materials and methods

### Immunohistochemical analysis

IHC was performed as described previously [26], with primary antibodies against CGRP (C9487, Sigma-aldrcich, America) and PGP9.5 (EPR4118, Abcam, America). Paraffin-embedded 3 μm-thick sections were deparaffinized, rehydrated and heated with citric acid buffer at 95 °C for 20 min for antigen retrieval. Sections were cooled and immersed in 0.3% hydrogen peroxide for 20 min to block endogenous peroxidase activity, rinsed in phosphate-buffered saline (PBS) for 5 min and blocked with 3% bovine serum albumin (BSA) at room temperature for 20 min. Tissues were incubated with the indicated primary antibodies in a humidified chamber overnight at 4 °C. After several washes with PBS, the sections were incubated with horseradish peroxidase (HRP)-labeled goat anti-mouse or goat ant-rabbit secondary antibody (Gene Tech; Shanghai, China) for 45 min at 37 °C. Diaminobenzene was used as the chromogen, and hematoxylin was used to counter stain nuclei. The sections were dehydrated, cleared and mounted. The staining intensity was classified into four categories: none (0), weak brown (1), moderate brown (2), and strong brown (3). The proportion of positive cells was determined by the analysis of image J (Rawak Software Inc., Stuttgart, Germany), and the positive aeras of staining was divided into five categories: none (0), 1–25% (1), 26–50% (2), 51–75% (3), 76–100% (4). The staining score was calculated by multiplying both scores. Staining scores below the mean value were considered to be “low expression,” and scores above the mean value were considered to be “high expression.”

### Patient samples

Preoperative plasma from 70 OSCC patients and 60 matched healthy donors was collected at the Department of Oral and Maxillofacial Surgery-Head and Neck Oncology, Shanghai Ninth People’s Hospital, Shanghai Jiao Tong University School of Medicine. None of the patients had received chemotherapy or radiation therapy prior to surgery. Fresh venous blood samples were collected from fasting subjects, stored in ethylene diamine tetraacetic acid tubes, and processed within 2 h. Eight OSCC tissues and paired adjacent normal tissues were obtained during surgery, immediately frozen in liquid nitrogen, and stored at − 80 °C until further analysis. The medical records of 218 OSCC patients were obtained from Shanghai Ninth People’s Hospital, Shanghai Jiao Tong University School of Medicine. A total of 84 paraffin-embedded primary specimens were collected at the Department of Oral Pathology of Shanghai Ninth People’s Hospital and a total of 74 patients were successfully followed up. TNM staging was based on the latest American Joint Committee on Cancer staging criteria [27].

### Cell culture

Human HN6 OSCC cells were kindly provided by the University of Maryland Dental School, USA. CAL27 cells were purchased from the American Type Culture Collection (USA). All cells were maintained in Dulbecco’s minimum essential medium (Invitrogen, Carlsbad, CA, USA) supplemented with 10% fetal bovine serum, 100 units/ml penicillin, and 100 μg/ml streptomycin and incubated in a humidified atmosphere with 5% CO_2_ at 37 °C.

### Western blot

Cells were harvested in RIPA lysis buffer (Beyotime, Haimen, China), and cell lysates were electrophoresed on 4–20% polyacrylamide gels and transferred to a polyvinylidenedifluoride (PVDF) membrane. Antibodies against the following proteins were used: CGRP (#14959, CST, USA), CLR (A8533, abclonal, USA) and GAPDH (#2118, CST, USA). The membranes were incubated with the appropriate primary antibody overnight at 4 °C and incubated with the appropriate secondary antibody (#7074, CST, USA) for 1 h at room temperature.

### ELISA assay

Fresh venous blood samples were drawn into ethylene diamine tetraacetic acid (EDTA) tubes from fasting subjects and processed within 2 h. The whole blood was centrifuged at 3000 round per minute for 15 min, and the supernatant was plasma and was extracted. The human plasma CGRP content is analyzed by human CGRP-1ELISA kits (E-EL-H0619c, Elabscience, Wuhan, China) according to manufacturer’s introduction.

### Clone formation and wound healing assays

These assays are well described in our previous research [28]. HN6 and Cal27 cells transfected with siCLR or negative control (siNC) for 24 h were seeded in six-well plates (1000 cells per plate) for 10 days. The cells were washed with PBS, fixed with 4% paraformaldehyde for 30 min, and stained with Coomassie brilliant blue for 30 min. Colonies with over 50 cells were identified as clones. For wound healing assay, 100% density of cells were cultured in six-well plates with serum-free medium, and cell monolayer was subsequently scratched with a 200-ul pipette tip. Representative images of cell migration were captured at 0 h, 12 h and 36 h after injury.

### Statistical analysis

All values are presented as means ± standard error of the mean (s.e.m.) in bar graphs and scatterplot diagrams. For box plots, center lines represent the median, limits represent the quartiles, and whiskers represent the minimum and maximum values. An unpaired Student’s *t*-test with no assumption of equal variance was used for comparisons between two groups. For comparisons of more than two groups, ANOVA (using a general linear model) was used. When the overall F test was significant (*P* < 0.05), post hoc comparisons using Tukey’s method of adjustment were used to assess the presence of any significant pairwise differences. Prognosis prediction performance was evaluated by the area under the ROC curve (AUC). The Youden index was calculated for the prognosis models. Overall survival was calculated using the Kaplan–Meier method. Univariate and multivariate survival analyses were conducted with a Cox regression model. Analyses were performed using GraphPad Prism 8 software (GraphPad Software Inc., San Diego, CA, USA). A two-sided *P*-value of < 0.05 was considered to indicate statistical significance.

## Results

### PNI was correlated with and predicted LNM in OSCC patients without taking T stage into account

**Fig. 1:**
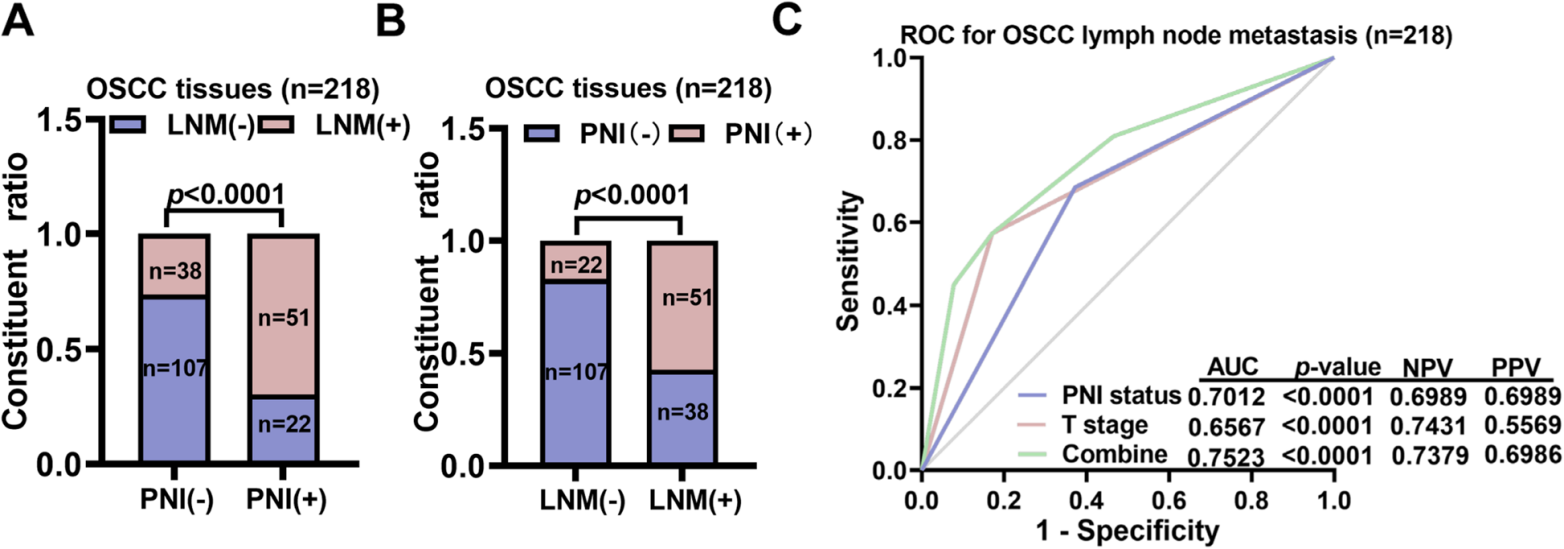
OSCC patients with PNI had a higher incidence of lymph node metastasis. **A** OSCC patients with lymph node metastasis (LNM(+)) were classified based on the presence or absence of PNI. **B** OSCC patients with PNI classified based on the presence or absence of LNM. **C** The PNI status yielded an AUC value of 0.7012 to predict LNM in OSCC patients, which was better than that obtained for the T stage. Both the PNI status and T stage were included in the multivariant logistic regression model to yield the combination value

We first retrospectively explored the correlation between PNI and the LNM status through a cohort of 218 OSCC patients diagnosed between 2014 and 2020 from Shanghai Ninth People’s hospital (Table 1). Patients with PNI had a higher incidence of LNM (51/73 [69.86%] vs. 38/145 [26.2%], *P* < 0.0001) (Fig. 1A). Patients with LNM had a high incidence of PNI (51/89 [57.3%] vs. 17.05%, *P* < 0.0001) (Fig. 1B). We subsequently determined if PNI status could be used to predict LNM. By ROC analysis, we found that PNI status yielded an AUC value of 0.7012 to predict LNM in OSCC patients, which was better than that obtained for the T stage. The sensitivity was 57.3% and the specificity was 82.95%, which was not considered satisfactory (Fig. 1C).

**Table 1:** Patient Demographics

Variable	No.(%)
**Age**	
≤ 60	116(53.2%)
> 60	102(46.8%)
**Sex**	
Male	143(65.5%)
Female	75(34.5%)
**T stage**	
T1/T2	109(50.0%)
T3/T4	109(50.0%)
**N stage**	
LNM (−)	129(59.2%)
LNM (+)	89(40.8%)
**Tumor location**	
Tongue	100(45.9%)
Cheek	31(14.2%)
Gingiva	34(15.6%)
Palate	23(10.6%)
Mouth floor	17(7.8%)
Mandible and maxilla	13(5.9%)
**Perineural invasion**	
PNI (−)	145(66.5%)
PNI (+)	73(33.5%)

### The neuropeptide CGRP correlated with both PNI and LNM

**Fig. 2:**
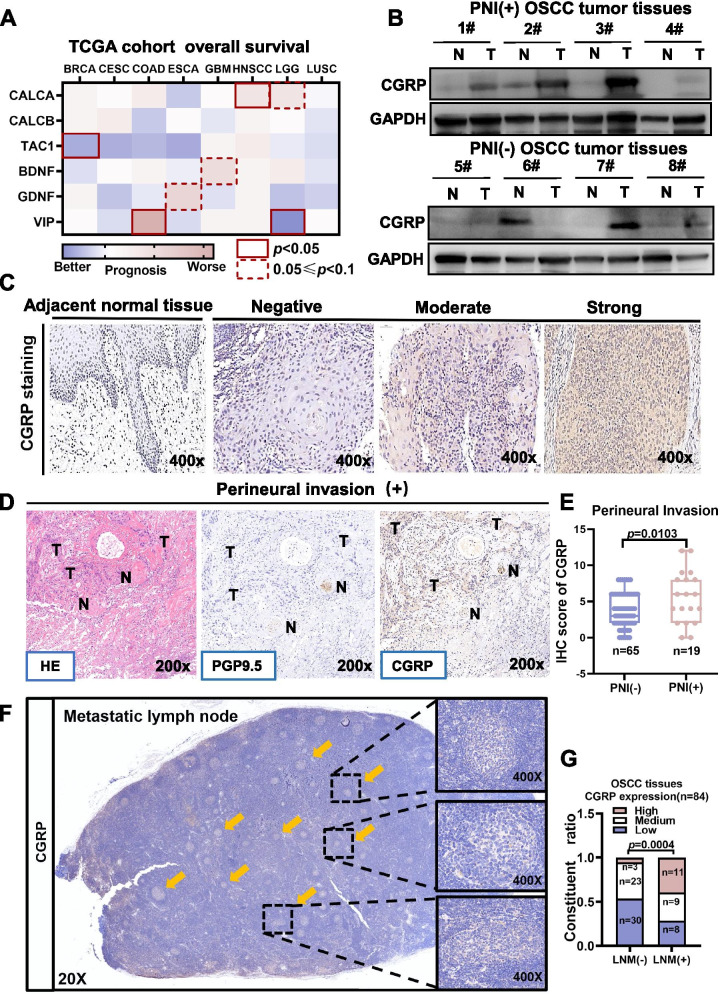
The nociceptor marker calcitonin gene-related peptide (CGRP) was correlated with both PNI and LNM in OSCC patients. **A** Kaplan–Meier survival analysis of cancer patients stratified by different neuropeptide mRNA levels according to the TCGA database. **B** Protein expression of CGRP in tumor tissues and adjacent normal tissues in PNI(−) and PNI(+) OSCC tissues. **C** Representative IHC images of OSCC tissues with different CGRP staining intensity. **D** Representative IHC images of PNI tissue from OSCC patients stained for CGRP and the nerve marker PGP9.5. **E** Quantification of CGRP scores in PNI(−) and PNI(+) OSCC tissues. **F** Representative immunohistochemistry images of a metastatic lymph node stained for CGRP. **G** CGRP staining levels in LNM(−) and LNM(+) OSCC tissues. BRCA, Breast invasive carcinoma; COAD, Colon adenocarcinoma; ESCA, Esophageal carcinoma; GBM, Glioblastoma multiforme; HNSCC, Head and Neck squamous cell carcinoma; LGG, Brain Lower Grade Glioma; LUSC, Lung squamouscell carcinoma

Despite the strong correlation between PNI and LNM, PNI status is usually determined after surgery through histopathological analysis, which limits its application. Due to the abundant innervation of nociceptive nerves branching from the trigeminal nerves in oral mucosa, we postulated that circulating neuropeptides released from nociceptors could potentially predict PNI status preoperatively. By mining the single-cell database of the murine nerve system (http://mousebrain.org/genesearch.html), we found that *Calca* (encoding CGRP-I) was the most abundant neuropeptide-encoding mRNA in dorsal root ganglia and trigeminal ganglia. We next explored the mRNA expression of several neuropeptides, including *Calca*, as well as *Calcb* and *Tac1*, which are considered to be coexpressed with *Calca,* in different tumor types according to the TCGA database (Fig. 2A). Of all these candidates, only *Calca* was correlated with poor overall survival in head and neck cancer (hazard ratio [HR] = 1.4, *n* = 583, *P* = 0.035) (Fig. 2A and S1).

Further, we measured protein levels of CGRP in OSCC tissues. Interestingly, in OSCC patients with PNI, expression of CGRP was higher in tumor tissues compared to adjacent normal tissues, while in patients without PNI, expression of CGRP was lower and similar between tumor tissues and adjacent normal tissues (Fig. 2B). This implied that CGRP could have been secreted from invading nerves.

To test this hypothesis, 84 OSCC tissues were subjected to IHC staining against CGRP and the nerve marker PGP9.5. We found that CGRP was co-expressed with PGP9.5 and showed strong staining at the perineural region (Fig. 2C-D). Quantitative analysis revealed that the OSCC tissues with PNI had a higher CGRP staining score in OSCC tumor tissues (*P* = 0.0103) (Fig. 2E). More importantly, we found that CGRP was stained in the metastatic niches of lymph nodes (Fig. 2F). Consistent with this, the quantitative analysis identified that OSCC tissues with LNM had higher CGRP expression in OSCC tumor tissues (*P* = 0.0004) (Fig. 2G).

**Fig. 3:**
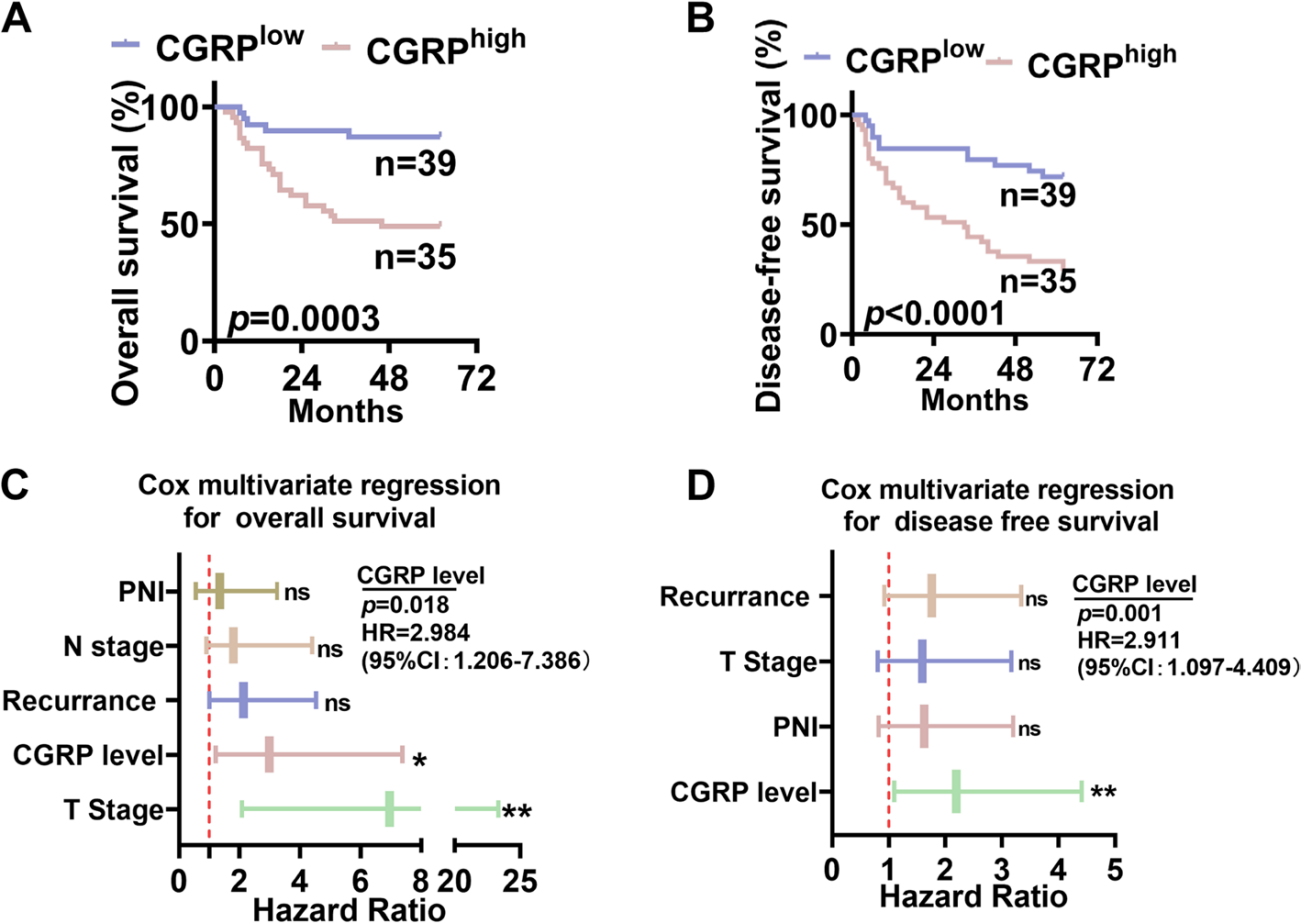
High CGRP expression in OSCC tumor tissues was correlated with poor overall survival. **A-B** Kaplan-Meier analysis revealed that OSCC patients with high CGRP expression in tumor tissues had worse overall survival rate and disease-free survival rate. **C** Multivariate Cox regression analysis revealed CGRPexpression in OSCC tumor tissues is an independent risk factor for overall survival in OSCC patients. **D** Multivariate Cox regression analysis revealed CGRP expression in OSCC tumor tissues was an independent risk factor for disease-free survival in OSCC patients

Furthermore, expression of CGRP in OSCC tumor tissues was an independent risk factor of both overall survival and disease-free survival rate in OSCC patients (Fig. 3A-D).

### Preoperative plasma CGRP levels predicted LNM in OSCC patients

**Fig. 4:**
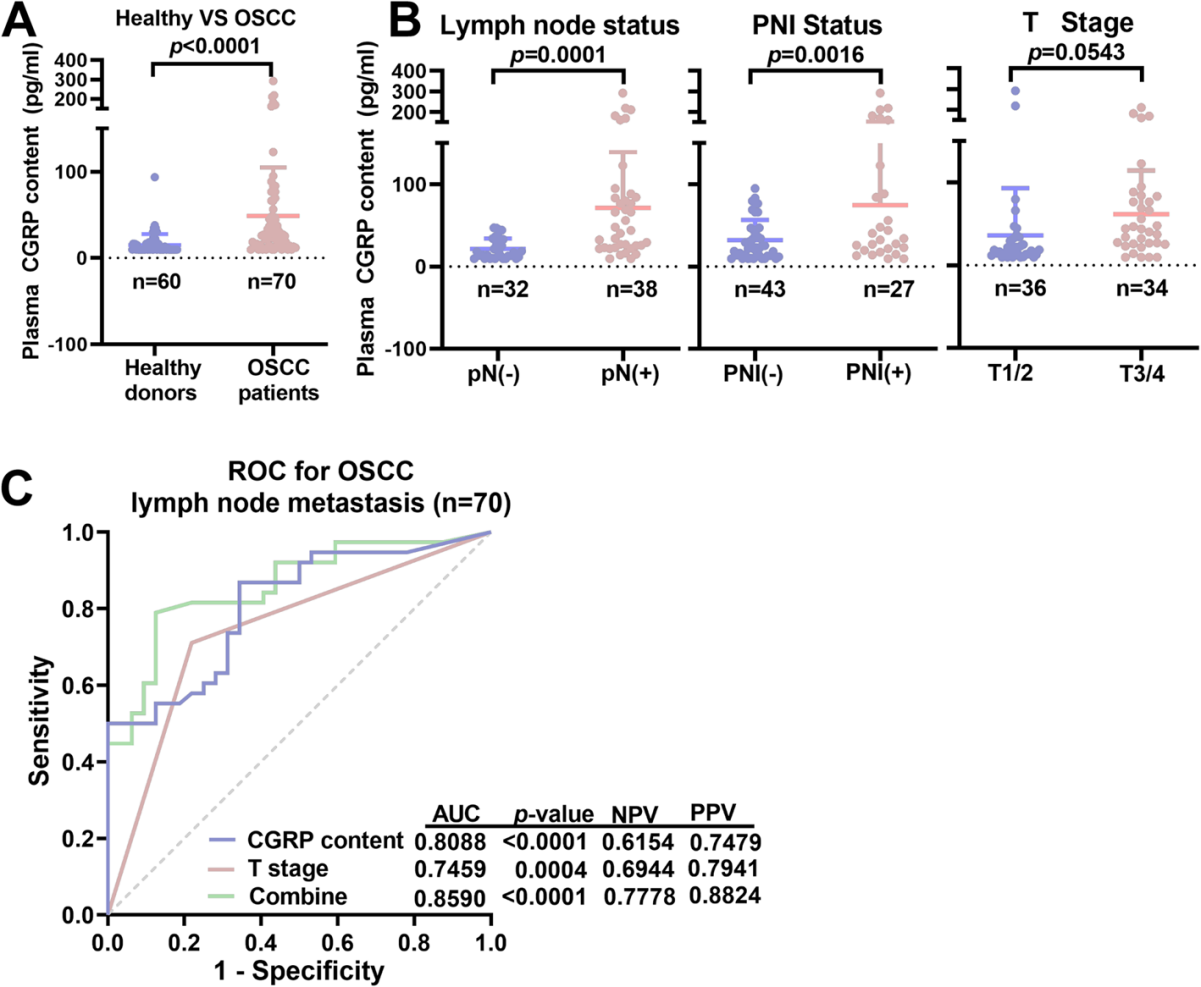
Circulating CGRP levels were predictive of LNM in OSCC patients. **A** Plasma CGRP levels in OSCC patients (*n* = 70) and healthy donors (*n* = 60), as analyzed by ELISA. **B** High CGRP levels were correlated with LNM, PNI, but not advanced T stage(T3/T4) in OSCC patients. **C** The combination of preoperative Tstage and plasma CGRP levels was calculated by multivariant logistic regression and yielded an AUC value of 0.8590 to predict LNM in OSCC patients

We found that CGRP expression in tumor tissues and metastatic lymph nodes was correlated with both PNI and LNM in OSCC patients, and that trigeminal ganglion neurons were pseudounipolar. Therefore, we postulated that substances with biological activity could be released in peripheral nerve endings once the nociceptive nerves are activated, and explored the feasibility of using peripheral CGRP as a preoperative marker to predict LNM. Peripheral blood from 60 healthy donors and 70 age- and sex-matched OSCC patients was collected, and plasma was extracted to measure CGRP levels by ELISA. Strikingly, CGRP levels were significantly higher in OSCC patients compared to healthy controls (48.59 vs. 14.98 pg/ml, *P* < 0.0001) (Fig. 4A). Among the 70 OSCC patients, CGRP levels were significantly higher in patients with LNM (*P* < 0.0001) and PNI (*P* = 0.0016), but not correlated with T stage (*P* = 0.0543) (Fig. 4B).

We then explored the predictive efficiency of preoperative plasma CGRP levels. ROC analysis revealed that CGRP alone yielded an AUC value of 0.8088, with a negative predictive value of 61.54% and a positive predictive value of 74.79% (Fig. 4C). The best cutoff value of CGRP levels was 21.66 pg/ml, which yielded a Youden’s index of 0.5247. Preoperative T stage was easy to determine based on clinical features and preoperative imaging. We found that the combination of CGRP and preoperative T stage yielded an AUC value of 0.8590 (*P* < 0.0001), with a negative predictive value of 77.78% and a positive predictive value of 88.24% (Fig. 4C).

Taken together, these findings demonstrated that preoperative plasma CGRP levels were correlated with LNM and PNI, and could therefore be used to predict LNM in OSCC.

### CGRP promoted differentiation of OSCC cells into malignant phenotypes in vitro through its receptor CLR

**Fig. 5:**
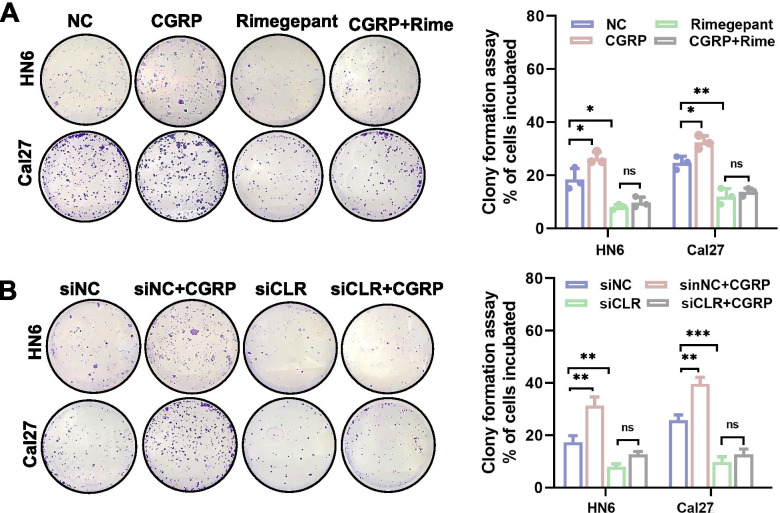
CGRP promoted the clone formation ability of OSCC cells in vitro through its receptorcalcitonin receptor like receptor (CLR). **A** CGRP promoted clone formation ability of OSCC cells, and blockade of CGRP receptor using CLR inhibitor Rimegepant antagonized the effects of CGRP in both HN6 and Cal27 cells. **B** Knockdown of CLR using small interfering RNA antagonized the effects of CGRP in both HN6 and Cal27 cells

**Fig. 6:**
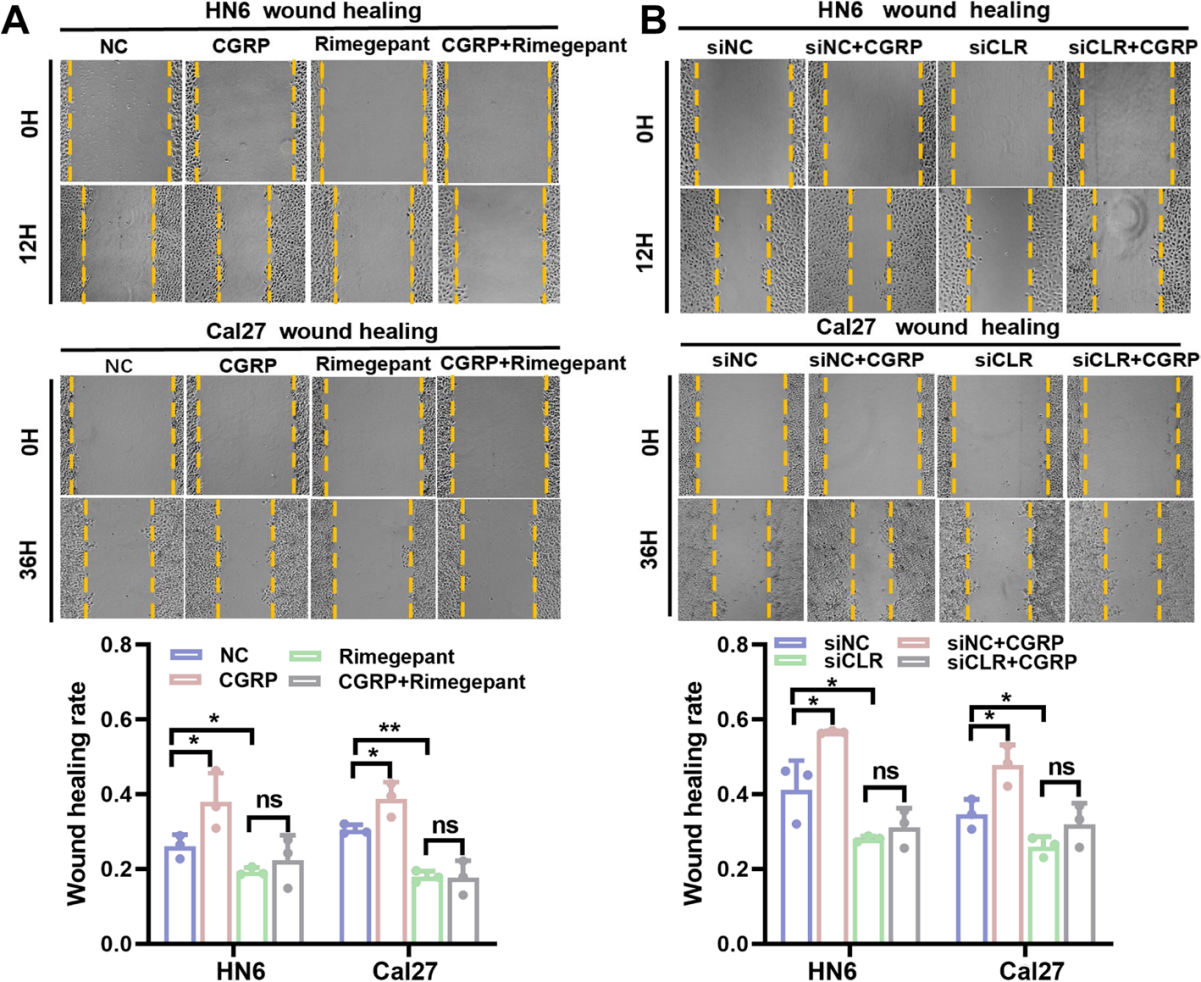
CGRP increased the wound healing ability of OSCC cells in vitro through itsreceptor calcitonin receptor like receptor (CLR). **A** CGRP increased the wound healing capacity of OSCC cells, and blockade of CGRP receptor using CLR inhibitor Rimegepant antagonized the effects of CGRP in both HN6 and Cal27 cells. **B** Knockdown of the CGRP receptor using small interfering RNA antagonized the effects of CGRP in both HN6 and Cal27 cells

Because CGRP was correlated with LNM and PNI in OSCC, we next investigated whether CGRP could influence the phenotypes of OSCC cells. Colony formation and migration are two essential functions for cancer cell metastasis. CGRP increased colony formation ability (Fig. 5A, B) and wound healing ability (Fig. 6A, B) of the human OSCC cell lines HN6 and Cal27. CGRP needs to bind with its receptor CLR (encoded by *CALCRL*) on cell membrane and transmit downstream signals. These promoting effects of CGRP could be antagonized by either treatment with the CGRP receptor antagonist Rimegepant or knockdown of the CGRP receptor CLR using small interfering RNA (siRNA) (Fig. 5A-B, 6A-B and S2).

## Discussion

In the present study, we found that PNI was correlated with LNM in OSCC. We further identified CGRP as a molecular link between PNI and LNM. Most importantly, preoperative plasma CGRP levels, combined with preoperative pain status and T stage, can be used to predict LNM.

As we found that OSCC patients with PNI had a higher incidence of LNM, several studies have reported that the PNI status was correlated with LNM in OSCC [29–31] . But the underlying biological connection between PNI and LNM is rarely reported. Nerves, as an important component in tumor microenvironment, has now emerged as a promising candidate [32, 33]. The oral cavity is densely innervated by the sensory nerves branching from the trigeminal nerves [34]. Prior studies have demonstrated the role of nerves in OSCC development. For example, Myers et al. reported that p53 loss led to the reprogramming of sensory nerves into sympathetic nerves, which promoted tumor progression [35]. A recent study demonstrated that lymph nodes are innervated by a unique population of sensory nerves [19]. These studies together indicate that nerves could connect different organs such as the oral mucosa and lymph nodes. If so, there may exist an anatomical and molecular connection between PNI and LNM in OSCC. Further studies should use in vivo models to verify the correlation of PNI and LNM via direct regulation of nerves.

Based on the clinical correlation between PNI and LNM, many researchers found that PNI can well predict the LNM [36]. However, the PNI status can be only determined after surgical dissection of tumor tissue, limiting its practical use in LNM prediction, and the preoperative non-invasive examinations are more attractive to clinicians to make personalized treatment. Preoperative blood tests are promising tools to predict survival and LNM in patients with malignant tumors such as colorectal cancer [37], renal cell cancer [38] and OSCC [39]. We found that preoperative plasma CGRP levels were predictive of LNM, yielding an AUC value of 0.8088. More importantly, the combination of preoperative plasma CGRP levels and preoperative T stage yielded an AUC value of 0.8590, providing a promising predictive model. It can partly be explained by that tumors with advanced T stages are not always companied with PNI. However, the correlation between preoperative plasma content of CGRP and the prognosis is unknown. Long-term follow-up is still required to answer this question.

CGRP, which plays a key role in migraine, has been reported to promote tumor progression by either directly affecting tumor cells [40] or promoting tumor-associated angiogenesis [25, 41]. More importantly, we found that the CGRP expression in OSCC tumor tissues was higher in OSCC patients with PNI or LNM and CGRP was found to promote the clone formation and wound healing abilities of OSCC cells. Due to that CGRP is often used as a marker of nociceptive nerves and is the most abundant neuropeptide in trigeminal nerves [20, 21], we think that the neurogenic CGRP may enhance the malignant phenotypes of cancer cell and lead to PNI and LNM. Further studies may use genetic modification mice to specifically regulate CGRP in nerves to verify this hypothesis.

## Conclusions

PNI and LNM are correlated in OSCC. The neuropeptide CGRP linked PNI and LNM in OSCC, and preoperative plasma CGRP levels could be used to predict LNM in OSCC patients.

## Supplementary Information

**Additional file 1.**

## Data Availability

All data generated or analyzed during this study are included in this published article.
