# Supplementary material for: The neuropeptide calcitonin gene-related peptide links perineural invasion with lymph node metastasis in oral squamous cell carcinoma
Source: BMC Cancer. 2021 Nov 20;21:1254. doi: 10.1186/s12885-021-08998-9 (PMC8606076; doi:10.1186/s12885-021-08998-9)
Supplement: Supplementary file 1 — Additional file 1. [file 12885_2021_8998_MOESM1_ESM.zip › Supplementary figures 2.0.pdf]

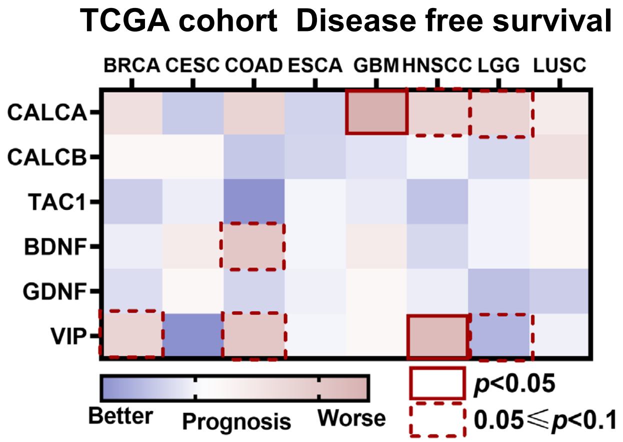

Supplementary Figure 1: Kaplan–Meier survival analysis of cancer patients stratified by different neuropeptide mRNA levels according to the TCGA database.

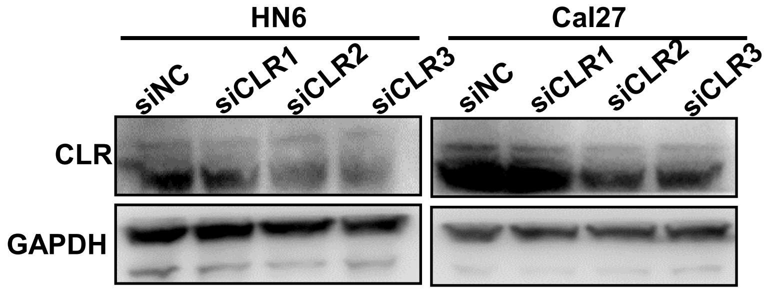

Supplementary Figure 2: The verification of knockdown of CALCRL in human OSCC cell line by siRNA.
