# Supplementary figures and images for: The neuropeptide calcitonin gene-related peptide links perineural invasion with lymph node metastasis in oral squamous cell carcinoma
Source: BMC Cancer. 2021 Nov 20;21:1254. doi: 10.1186/s12885-021-08998-9 (PMC8606076; doi:10.1186/s12885-021-08998-9)

**Figure 2B**

**CGRP**

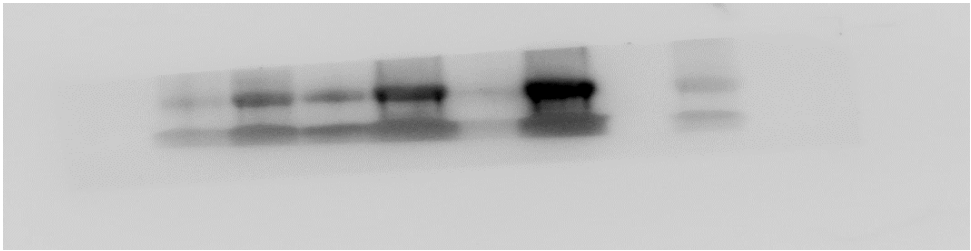

**GAPDH**

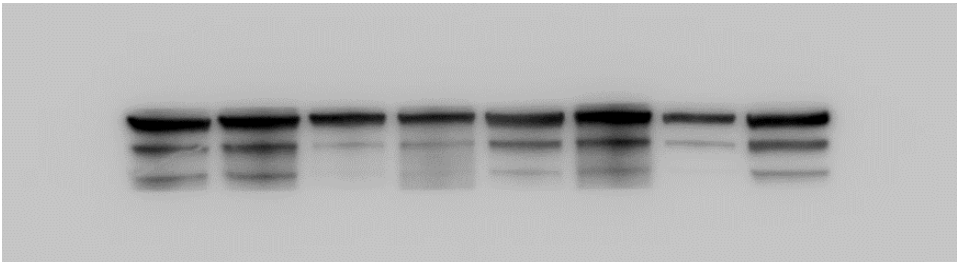

**CGRP**

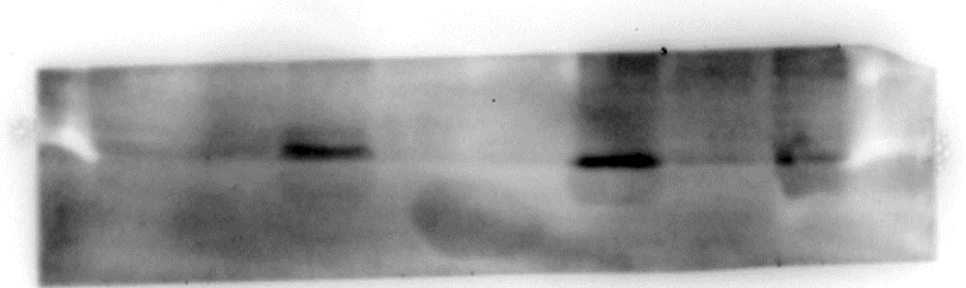

**GAPDH**

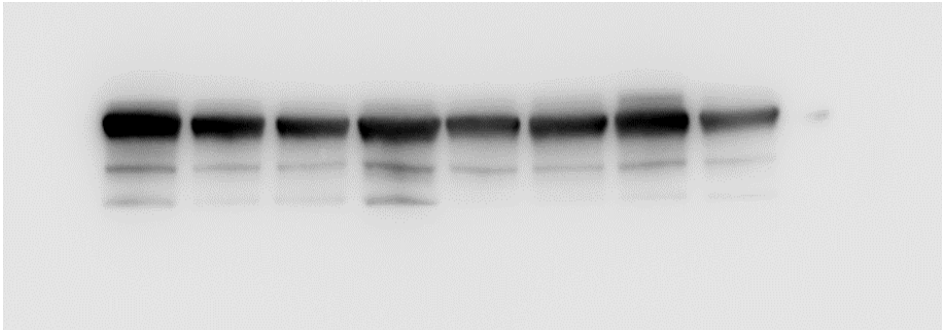

**Supplementary Figure 1B**

**CLR**

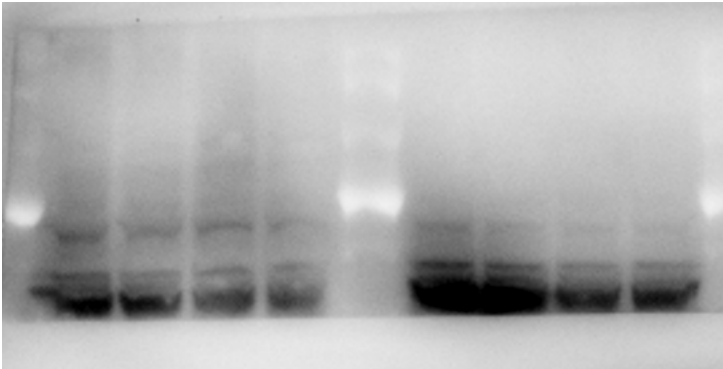

**GAPDH**

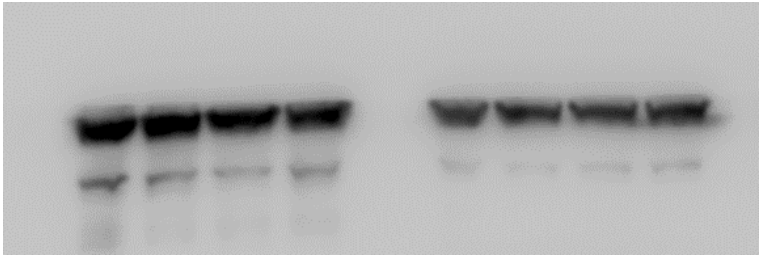

Supplement: Supplementary file 1 — Additional file 1. [file 12885_2021_8998_MOESM1_ESM.zip › Unprocessed gels.pdf]
